# Hospital Festivals and Meetings

**Published:** 1894-05-12

**Authors:** 


### HOSPITAL FESTIVALS AND MEETINGS.

### THE SIR ANDREW CLARK MEMORIAL.

The meeting held in Princes' Hall on May 3rd, in connection with the proposed memorial to the late Sir Andrew Clark, will long live in the memories of those who were fortunate enough to be present at it. Hours before the time announced for its commencement, an orderly crowd covered the pavement, pressing so closely round the entrance-doors that the possessors of admission tickets had some difficulty in getting near enough to present them. The hall was soon filled in every part, for people without tickets were admitted until every seat, and almost every inch of standing room, was taken up. Patients, old and young, of the well-beloved physician thronged in, and past and present workers from the London Hospital, that great institution to which Sir Andrew always thought he owed so much of his success, and to which he gave in return his skill and his time with lavish generosity.

The accommodation for the reporters seemed great, but the attendance of "the gentlemen of the Press" was extraordinarily large, and they could not all find seats. The whole assemblage bore the aspect of "a special occasion," and the eager interest of all present in Princes Hall rendered the gathering markedly different from many afternoon assemblies, for boredom was absent, and every one came because they liked and not because they thought they ought to be present.

A whisper went round that punctual to time Mrs. Gladstone had come in and taken her seat, and few could forget that she had discovered and appreciated the talent of "that great Andrew Clark," whom she found enthusiastically working amongst the sick poor of Whitechapel, for whom her sympathy was claimed. The applause which greeted the appearance of Mr. Gladstone on the platform a few minutes later was heart-stirring indeed, and yet withal it had pathos. It was so evident that a great effort had been made by him to bear a part in this gathering, yet it was doubtless an effort willingly made for a good cause, and one very dear to the heart of Mr. Gladstone's late friend and physician.

We publish Mr. Gladstone's speech in full elsewhere.

The Duke of Cambridge, Cardinal Vaughan, Mr. Jonathan Hutchinson, Canon Wilberforce, Sir James Paget, Mr. J. H. Buxton, and Mr. Ernest Hart were the other speakers, and it was noticeable that nearly all of them had some personal reminiscence of Sir Andrew, and that they invariably showed some liberal or kindly feature of his character.

It is proposed that the memorial should take the form of a block of buildings containing small wards for the isolation, observation, and treatment of certain cases always liable to obtain admission, or to develop in those under treatment in a general hospital. Such cases, being of an infectious nature, cannot with safety to the main body of patients be treated in the ordinary wards without danger to others, or if sent away to a fever hospital they are exposed to fresh dangers to themselves. These wards would not be used for the admission of infectious cases or of fever cases, so that the number of occupied beds in the hospital or the cost of maintenance would not be materially increased. It is further hoped that these buildings may be so arranged as to contain the complete pathological department, the present arrangements of which, though well suited for the hospital before it reached its present importance, are quite inefficient for the existing needs of the institution. Sir Andrew Clark was closely identified with this important department during the earlier years of his office as assistant-physician at the hospital, and his teaching throughout shows the great importance which he attached to the study of pathology as a training for medical practice. It is earnestly hoped that all who have had reason, either as friends or patients, for gratitude to, or regard for, Sir Andrew Clark, will show their appreciation of his many great qualities by liberally contributing to this fund. The establishment of the proposed memorial will not only tend to preserve his name in high honour, but will have far reaching results in the relief of suffering, and the advancement of medical science. The total cost of the proposed building is estimated at £12,000. The payment of subscriptions may be spread over three years.

---

### KING'S COLLEGE HOSPITAL DINNER.

The fine roomy hall of Lincoln's Inn was well filled a week ago Wednesday night by some 200 guests at the King's Colleg Hospital Festival Dinner. The institution owed to the kind permission of the Masters of the Bench their meeting in so appropriate and convenient a place, but the interest of the legal profession in the hospital is well known, arising as it does out of near association and close sympathy, and much of the support which enables this branch of philanthropic work in Portugal Street to be carried on under more than usually favourable circumstances comes from past and present wearers of wig and gown. For, after all is said and done, the hospital, although its work is very large and wide-spreading, has cause to congratulate itself upon its position. It does not appeal for assistance to clear off a debt, but rather for help to make a yearly income less precarious, and to avoid, if possible, the undesirable course of reckoning legacies as income for the year in which they are paid. The Right Hon. Lord Justice Davey occupied the chair, and the company present included:

The example of brevity was set by the Chairman in proposing the Royal toasts, and he then gave the toast of the evening, "Success to King's College Hospital." The Hospital Committee, he said, found themselves this year compelled to make a special and more urgent appeal than usual, though during the past year, notwithstanding the depression in trade and the shrinking of securities, the subscriptions had been maintained at a most creditable figure. They had in the hospital 220 beds, of which on the average about 200 were continously occupied; the number of in-patients treated last year was 2,372, and the number of out-patients in round numbers 24,000. The expenses of the institution were rather more than £19,000, and as to their income they had £1,000 from securities, and a small sum arising from casual receipts—nursing and so forth—but with these exceptions they were absolutely dependent upon the charity of the benevolent public. He thought he was right in saying that, taking the subscriptions and other sources of income at their respective normal amounts, they had an income of about £10,000 a-year. Consequently, in the past, they had been obliged to expend the sums received in the form of of legacies, but these they wished instead to turn into a permanent fund for the benefit of the hospital. The legacies amounted to something over £6,000 a-year, and those interested in the hospital were endeavouring to raise £50,000 to form a maintenance fund. The total amount promised was up to the present £7,669, of which £5,178 had been received, and by transferring £3,700 to the current maintenance provision, the total income was thus raised last year to £19,000. But it was obvious that legacies were a precarious source of income, and the committee would be very glad to increase their permanent endowment by these contributions, rather than consume them in the year. They had now a permanent staff of seven qualified resident medical and surgical officers and 79 sisters, nurses, and probationers. After pointing out that contributors to King's College Hospital could be sure that, in these days of imposture and professional mendicancy, they could not do any harm and must be doing much good, the Chairman said that the institution existed not only for relieving the medical and surgical needs of the poor but was also a great school of medicine, and he could imagine nothing more useful or more necessary than the giving of adequate support to the school in which doctors were trained to attend the sick. He thought that the nursing staff of the hospital would compare favourably with that of any similar institution in London, and he was charged that night with a message from Lady Bowen. In his last hour Lord Bowen was nursed by nurses from King's College Hospital, and Lady Bowen said she could not speak too warmly of their sympathy, their kindness, and their activity. (Applause).

The Rev. Dr. Wace acknowledged the toast on behalf of the hospital, saying that there was no institution in London or elsewhere which supported King's College Hospital with so sustained a generosity as the Honourable Society of Lincoln's Inn. The hospital stood at the present time in as satisfactory a position as institutions in dependence on voluntary contributions could expect to be, for they could never look much ahead. All persons connected with the hospital were working together in harmony and co-operation, and the institution supplied the four things that such an organisation should present to the public: (1) Foremost hat it should relieve the poor; (2) that it should form a great school for the advancement of medical science; (3) that it should be a great school for the medical education of young men; and (4) that the work should be done with economy. Other toasts followed.

The dinner proved so far successful that subscriptions were announced amounting to £2,200.

### HAMPSTEAD HOME HOSPITAL.

On May 2nd the biennial festival dinner of the Hampstead Hospital was held at the Langham Hotel, Mr. H. Harben presiding. Subscriptions and donations amounting to £1,463 were announced by the hon. secretary during the evening. This sum included a donation of £550 from the chairman.

### FESTIVAL DINNERS OF HOSPITALS FOR CHILDREN.

Two very interesting festival dinners in connection with hospital work among children have taken place within the past few days. The friends and supporters of the Great Ormond Street institution met at the Hotel Metropole on Saturday night, and at the same place on Monday there was held the annual reunion of the equally useful, though much less well-known organisation in Shadwell. Coming as they did in such close sequence, these two festivals, which externally differed so much, presented a very valuable lesson in metropolitan hospital work. They once more demonstrated how great is the demand for efforts of this kind, how late the beginnings, how inadequate the measure of relief, and yet how zealous is the prosecution of labours in this cause by those who really appreciate the gravity and necessity of the work, and remember too, that perhaps if we were able to do more, medically at least, for the children of our great city, we might have less need to care for them in after years at the great institutions for adults. In treating these dinners, and with them the work of the hospitals from a common point of view, it is especially desirable to draw no comparison, which in any case under the circumstances could serve no useful purpose, though few people can fail to be struck with the disproportion of the support accorded in the two casess for the difference in regard to the scope of labour, cannot be held entirely accountable. There is no doubt that financial support was invited for the Great Ormond Street institution under unusually favourable circumstances, and was aided by individual efforts of no mean kind, yet as periods of special help do come round to most hospitals at times, if but rarely, one hopes the merits of the equally commendable and hard-working East-end organisation will soon be better recognised. Yet in neither case is there any need for despondency or even regret. If the springs of charity gradually dried up, the hospitals latest to draw life and health from them would be those for children, and all the speeches at both festivals breathed both encouragement and hope. Royalty patronised the dinner of the Great Ormond Street Hospital in the person of the Duke of York, who presided, and whose presence, doubtless, helped to attract much of the large and influential company, which included the Duke of Fife, Lords Sandhurst and Kinnaird, the Right Hon. George Denman, the Hon. Louis Greville, Rear-Admiral E. Hobart Seymour, General Lord Roberts, Sir Horace Farquhar, Mr. Justice Kekewich, Sir Wm. Broadbent, Sir Richard Quain, Hon. W. W. Astor, Mr. Alderman and Sheriff Dimsdale, Mr. George Newnes, M.P., Sir Reginald Hanson, M.P., Mr. Arthur Lucas (chairman of the hospital), Mr. E. Ponsonby, Mr. Robert Vyner, Mr. John Murray, Mr. Henry Arthur Jones, the Hon. Spencer Joliffe, Colonel Gordon Watson, Mr. A. Beit, Mr. Alfred Hoare, the Hon. Evelyn Hubbard, Lieut.-Col. F. Gore, Mr. G. Herring, Dr. Barlow, Dr. Sturges, and Mr. Edmund Owen. In responding to the toast of the "Prince and Princess of Wales and the other members of the Royal Family," the noble Chairman bore testimony to the great interest which his parents had always taken in the welfare of the hospital, his mother having laid the first stone of the present building in 1874, and having, together with his father, opened the new wing in June last year. His Royal Highness then proposed the "Army, Navy, and Reserve Forces," a toast which found exceptionally appropriate acknowledgers in Lord Roberts, with his easy conversational style, and Admiral Seymour, who made a thoroughly business-like oration. Then came the toast of the evening, which the Chairman proposed clearly and slowly without one moment's hesitation, and with much felicity of expression. He said: I have now great pleasure in proposing the toast of the evening, which is "Success and Prosperity to the Hospital for Sick Children," and I am sure it will meet with your hearty and cordial support. (Cheers). In bringing the claims of this hospital to your notice I will mention most briefly the principles on which it is governed, and the objects for which it was established. The principle is that poverty and sickness combined are the only qualifications necessary to obtain an entrance within its walls, and the objects which it seeks to attain are (1) the medical and surgical treatment of poor children; (2) the attainment and diffusion of knowledge regarding the diseases of children; and (3) the training of nurses for sick children. It should also be mentioned that this hospital is the parent of all other children's hospitals, as it was the first children's hospital established in Great Britain. It commenced its career in the year 1852, and during the 42 years of its existence no less than 600,000 children had passed through its wards. But in the last few years it was found to be absolutely necessary to make extensive additions to the existing building. More cots were required, the accommodation for the working staff was quite inadequate, and the out-patient department was far too small and very inconvenient. By the new building recently erected and opened last year 68 cots were added, the out-patient department was enlarged and remodelled, and by further additions for the working staff the committee are enabled to administer the affairs of the hospital far more efficiently and with greater economy. But the cost of this necessary building was very heavy, and there remains still the sum of £9,300 unpaid, and to this must be added the further amount of £2,000, the deficit on working expenses over income during 1893-4, making a total of £11,300 in all. It is to wipe off this debt that I ask your generous support to-night. In making this appeal, I do not seek to excite your sympathies by dwelling on the sickness and disease which has been treated, or the pain and suffering which have been alleviated during the past forty-two years—I could do this without any difficulty by telling you of some of the cases I saw only the other day when I was on a visit to the hospital—but I do ask you and the country to give support to this noble and useful institution, because of the good work it has done in the past, and which, with God's help, it will do in the future to the most helpless among us—little suffering children. (Cheers.)

The Duke of Fife, who is the president of the hospital, thanked the Duke of York, for aiding by his presence and eloquence, a truly noble charity, and saying that he had visited in great detail every portion of the institution, and had spoken to a considerable number of the little patients. His Grace then proposed the health of the "Committee of Management," to which Mr. Arthur Lucas responded, saying that they had a hospital which was capable of a vast amount of good. They had one addition to make, and then he trusted they would have a building which was perfection itself. That addition was to the north block, where such diseases as diphtheria, scarlet fever, and measles were treated in the most inadequate manner, as far as accommodation was concerned. He had, however, to announce that two friends of the hospital, the Misses Cohen, had expressed a willingness to defray the cost of the extension of the north block in memory of a deceased relative, the late Countess of Rosebery, who in her too short life had done so much to benefit the nursing of sick children. The proposal of "The Medical Officers," the last toast of the evening, gave Mr. H. A. Jones an opportunity for deprecating the tendency to treat pathological subjects on the stage, and at the same time to pay a really eloquent tribute to the medical profession, claiming that it was the kindest, the most tender, and the most humane of all professions. The toast was acknowledged by Dr. Sturges, the senior physician, and then came the eminently gratifying announcement that the very large sum—a record collection of the kind—of £10,066 had been subscribed, which included 25 guineas from the chairman, and 25 guineas from the Duke of Fife in addition to his annual donation of 20 guineas.

HOSPITAL FESTIVALS AND MEETINGS—*continued*

### EAST LONDON HOSPITAL FOR CHILDREN.

The dinner of the East London Hospital for Children was as already announced less assuming, but equally interesting. The Marquis of Zetland, who has made himself thoroughly conversant with the needs of the institution, occupied the chair, and among the company which on this annual occasion always includes ladies, were Lieut. Col. the Right Hon. Earl of Erroll, Sir Evan and Lady Smith, Mr. H. W. Trinder, Mr. and Mrs. C. Prescott and the Misses Prescott, Mr. F. Wootton Isaacson, M.P., Mr. W. H. Myers, M.P., Mr. C. Cheston, Mr. Joseph Spencer, Mr. H. C. Burdett, Revds. E. Bray, Father Gorman, A. R. Carter and J. Kennedy, Miss Samuelson, Mrs. Sydney Grundy, Mr. Louis A. Dunn, Dr. Dawson Williams, Mr. H. N. Custance, Major and Mrs. Roper Parkington, Dr. Eustace Smith, Mr. and Mrs. H. Betham, E. Robinson, Dr. Druce Slater, Dr. H. B. Donkin, and Miss Davies (lady superintendent.) The Royal toasts having been honoured, the Chairman proposed "The East London Hospital for Children," and, in dealing with a personal visit which he recently paid to the establishment, said that owing to its geographical position, it was not placed so as to catch the eye of the opulent or the wealthy. It was situate in the heart of the city, and was doing its good and useful work in an unostentatious manner in the densest of populations. During the year 1893 there were 1,533 in-patients, of whom 682 were discharged cured, 311 discharged relieved, 297 died, 148 were detained pending admission into fever hospitals, and 95 remained in the hospital on December 31st last. In glancing at the figures, do doubt it struck one that the mortality was high. It was very easily accounted for by the position of the hospital, though he was not prepared to say whether this was unique, by the fact that the institution was almost—he was not quite sure whether it was not absolutely—unique in receiving infants from birth without any restriction whatever. The new building for out-patients, opened some two years ago, had very materially benefited the working of that department, but there was still a debt on it. Yet he ventured to hope that before long funds might be forthcoming for the further erection of building accommodation for casualty patients. In the past year 9,568 out-patients had been received and 18,318 casualty patients treated, making a total of 27,886, or an increase on the numbers of the previous year of 2,272 and 6,037 respectively. In appealing to those present to support that most excellent institution, he asked them to take their part in relieving those miseries to which he feared many of the patients were necessarily born, and to subscribe to an institution which took so great a part not only in alleviating their present sufferings, but in fitting them to fill their future positions in the noontime or the eve of their lives. (Cheers.)

In replying to the toast, Mr. H. W. Trinder reviewed the work of the hospital since its foundation by Dr. Heckford, and pointed out that they received assistance of a very cosmopolitan order. He thought that the only organisation that could in any way compare with the East London Hospital in reaching the poorer classes was the one over which General Booth presided, of whom he desired to speak with honour. He was very glad to say that, through the energy of his predecessor in the chair of that hospital, Mr. Charles Cheston—(applause)—they had amassed, even in the present bad times, a fund of over £3,000—not promised, but actually paid—with which they intended during the coming year to purchase the land for and build a convalescent home upon the East Coast—at Clacton-on-Sea—which would enable them to send to the pure air, and keep there, far from their poor homes, until they really had become strong, children who came to their hospital, and who otherwise would leave it well, as far as medical science was concerned, but in a condition in which they could very easily relapse. After referring to the fairly satisfactory nature of their finances, which was much helped by a legacy at the close of the year, the speaker said they had also had another satisfactory legacy, which had enabled them to invest some money with the object of providing for a rainy day, and of providing an income with which to meet their largely increasing work. (Cheers.)

### THE RELIGIOUS QUESTION AT THE GERMAN HOSPITAL.

A VERY crowded meeting of governors of the German Hospital was held in the Great Hall of the Cannon Street Hotel on Tuesday last, to consider the vexed religious question at this institution. The special general court had been called by certain governors to consider the following resolutions: (1) The committee of the German Hospital having confirmed at their meeting held on March 8th, 1894, a resolution to the effect that the German Hospital is a Protestant institution, it is hereby resolved by this special general court that this resolution of the committee is: (*a*) Not in accordance with the idea which led to the founding of the German Hospital; (*b*) not in accordance with the opinion of the majority of the subscribers to the funds of the German Hospital; (*c*) contrary to the rules and regulations of the German Hospital. (2) That the German Hospital is and shall remain a national German institution, and shall be conducted on unsectarian lines, and strictly according to provision of Rule XXVI. (3) That in accordance with the previous resolution, religious services, instructions, or Scripture readings shall not be publicly held within the wards. Baron von Schröder, the treasurer of the hospital, and a vice-president, occupied the chair, but an attempt being made to oust him (on the grounds that the position should be occupied with perfect impartiality) in favour of Mr. G. Chambers, the motion was put to the meeting affirming that the Baron should leave the chair. After a good deal of noise the motion was easily defeated and the Baron continued to preside. The Rev. Father Verres then moved the first resolution, tracing the foundation of the hospital from its source. The institution, he said, was founded with the idea that it should be conducted with absolute fairness, justice, and equality to everyone therein, and the statement of Baron Schröder at the annual general court in January last that it was a Protestant institution had come as a surprise to the great body of subscribers. The rules simply said that the Protestant chaplain should visit the Protestant patients, and that no Scripture readings should be held within the walls, and though other denominations did not object that the nursing should be in the hands of Protestant deaconesses, they did object that one denomination should have the opportunity of holding public services in the wards which other patients holding different religious views were obliged to hear. The Lutheran Church adjoined the hospital, so the Protestant patients could easily attend service there. Mr. M. Kleimenhogen, in seconding the resolution, said the German Hospital should be a national hospital, and all creeds treated alike. Protestant Scripture-readings, however, were held in the wards, the Communion was administered openly—a statement which was vigorously denied by the Chairman, who affirmed that it was only administered privately to the ill or dying—tracts were freely admitted, sold, and distributed, the nursing staff had endeavoured to persuade Jewish patients to change their religion—this was also denied by Baron Schröder—and a member of the honorary medical staff, Dr. Ludwig, had testified to a prejudice against the Jews who wished to be treated, on which occasion a committeeman had said that patients of this belief were troublesome, dirty, and mentally and morally reduced. He (Mr. Kleimenhogen) admitted that, from their origin and surroundings, this might be the case with certain Jews, but surely those persons were the more deserving of sympathy. Mr. F. D. Mocatta appealed to those present to take away from the hospital its reputation of being a Protestant institution. Let there be a multiplication of chaplains of the various denominations, if necessary, but no proselytising. He had always understood that the German Hospital was open to all speaking the German tongue, and this definition excluded Austrians and Swiss, who in many cases were Catholics. Mr. Zimmerman contended that a hospital should not be a missionary station, and Mr. George Chambers also supported the resolution. Mr. Louis Davidson, chairman of the Visitation Committee, testified that no proselytising took place in the wards as far as the Jewish patients were concerned, and the general treatment they received was good, but as such patients were 15 per cent. of the whole, he thought, that ministers should be placed on the same footing as regards private ministration, and should not be allowed to hold any kind of service in the wards. Mr. S. Samuel suggested that a sub-committee should be appointed to draw up rules and regulations so that the various patients might enjoy to the full the ministrations of their respective pastors. Mr. W. J. Thompson said that the committee had legal advice that the chaplain was within his rights in holding service in the wards, and that the Lutheran pastor could not visit all the patients personally. The service was held twice a week. It lasted only from ten to fifteen minutes, and was of simple character, and not objected to even by those who did not hold Protestant views, and who usually only occupied some 25 beds out of the 125 in the hospital. He moved as an amendment to the resolution: "That the German Hospital is and shall remain a national German institution open to all sick poor speaking the German language without distinction of nationality or creed, and furthermore, that the committee be requested to conduct the internal management of the hospital as they have hitherto done." Mr. A. J. Allen seconded the amendment. The Chairman, in putting the amendment, and stating that he regarded the matter as a question of confidence, defended the innocent nature of the services, and pointed to the similar practice prevailing elsewhere of holding them, and further contending that there were no complaints from patients that they had been inconvenienced by these ministrations. The amendment was carried by 175 to 138, and adopted as a substantive resolution by 153 to 127 votes. The counting was difficult, and created great confusion; but the Chairman declined to take a division, though he offered a poll. This was refused, so after a few moments' delay, in which the turmoil increased, and insinuations were flung broadcast, Baron Schröder abruptly remarked, "the meeting is closed," put on his hat and left the room. This effectually terminated the proceedings, but the supporters of the original resolutions displayed much dissatisfaction.

